# Variation in ligand responses of the bitter taste receptors TAS2R1 and TAS2R4 among New World monkeys

**DOI:** 10.1186/s12862-016-0783-0

**Published:** 2016-10-12

**Authors:** Kei Tsutsui, Masahiro Otoh, Kodama Sakurai, Nami Suzuki-Hashido, Takashi Hayakawa, Takumi Misaka, Yoshiro Ishimaru, Filippo Aureli, Amanda D. Melin, Shoji Kawamura, Hiroo Imai

**Affiliations:** 1 Primate Research Institute, Kyoto University, Inuyama, Japan; 2 Department of Integrated Biosciences, Graduate School of Frontier Sciences, The University of Tokyo, Kashiwa, Japan; 3 Japan Monkey Centre, Inuyama, Japan; 4 Department of Applied Biological Chemistry, Graduate School of Agricultural and Life Sciences, The University of Tokyo, Tokyo, Japan; 5 Research Centre in Evolutionary Anthropology and Palaeoecology, Liverpool John Moores University, Liverpool, UK; 6 Instituto de Neuroetologia, Universidad Veracruzana, Xalapa, Mexico; 7 Departments of Anthropology & Archaeology and Medical Genetics, University of Calgary, Calgary, Canada

**Keywords:** Bitter taste receptor, TAS2R, G protein-coupled receptor, New World monkey, Interspecific functional variation, Molecular evolution

## Abstract

**Background:**

New World monkeys (NWMs) are unique in that they exhibit remarkable interspecific variation in color vision and feeding behavior, making them an excellent model for studying sensory ecology. However, it is largely unknown whether non-visual senses co-vary with feeding ecology, especially gustation, which is expected to be indispensable in food selection. Bitter taste, which is mediated by bitter taste receptors (TAS2Rs) in the tongue, helps organisms avoid ingesting potentially toxic substances in food. In this study, we compared the ligand sensitivities of the TAS2Rs of five species of NWMs by heterologous expression in HEK293T cells and calcium imaging.

**Results:**

We found that TAS2R1 and TAS2R4 orthologs differ in sensitivity among the NWM species for colchicine and camphor, respectively. We then reconstructed the ancestral receptors of NWM TAS2R1 and TAS2R4, measured the evolutionary shift in ligand sensitivity, and identified the amino acid replacement at residue 62 as responsible for the high sensitivity of marmoset TAS2R4 to colchicine.

**Conclusions:**

Our results provide a basis for understanding the differences in feeding ecology among NWMs with respect to bitter taste.

**Electronic supplementary material:**

The online version of this article (doi:10.1186/s12862-016-0783-0) contains supplementary material, which is available to authorized users.

## Background

Among the five basic taste qualities (sweet, bitter, sour, salty, and umami), bitter taste is thought to be particularly important for the survival of animals because it contributes to the avoidance of potentially toxic substances in food. Bitter taste is mediated by bitter taste receptors (TAS2Rs) [1, 2], which are mainly expressed in taste buds in the tongue. The repertoire of TAS2Rs in the genome varies considerably among animal species [3]. TAS2Rs are G protein-coupled receptors that recognize a wide variety of bitter substances as ligands.

The responses of human TAS2Rs to various bitter substances have been thoroughly investigated [4]. However, little is known about variation in the response properties of TAS2Rs among species. It is reasonable to speculate that the properties of bitter taste reflect adaptation to species-specific feeding environments. Variation in the response sensitivity of TAS2R16, a well-studied TAS2R, to salicin and its derivatives has been observed among humans, chimpanzees, and macaques [5, 6]. However, little is known about interspecific functional variation in TAS2R orthologs other than TAS2R16 and 38 in primates.

Among TAS2Rs, TAS2R1 and TAS2R4 are promising targets for interspecific comparative studies because they are relatively highly conserved among a wide range of species [3] and some functional studies have been performed [7–9]. In this study, we investigated interspecific variation in TAS2R1 and TAS2R4 in primates.

New World monkeys (NWMs) exhibit remarkable interspecific variation in color vision and diet (including fruits, nuts, leaves, flowers, plant exudates, and insects) [7–9], making them an excellent model for studying the relationships among senses [7]. For example, owl monkeys are nocturnal, lack cone-based color vision [8], and have primarily frugivorous diets supplemented with insects, flowers, nectar, and leaves [9]. Marmosets are polymorphic with respect to color vision (dichromacy in males and some females and trichromacy in other females) [10] and some species (e.g., *Callithrix jacchus*) have unique exudativore (gum-feeder) diets supplemented with insects [9].

In the present study, we performed calcium assays to compare the responses of NWM TAS2R1 and TAS2R4 to various bitter substances among species. We found that owl monkey TAS2R1 has a high sensitivity to camphor and marmoset TAS2R4 has a high sensitivity to colchicine. Furthermore, we inferred the amino acid sequences of a set of ancestral receptors of NWM TAS2R1 and TAS2R4. A functional assay of the ancestral receptors revealed the evolutionary timing of the shift in the ligand sensitivity of NWM TAS2R1 and TAS2R4. We also identified candidate amino acid residues responsible for the evolutionary shifts in ligand sensitivity. Furthermore, using site-directed mutagenesis experiments, we demonstrated that one of the candidate residues, F62, is responsible for the high sensitivity of marmoset TAS2R4 to colchicine. These results suggest a possible mechanism by which NWMs adapt to species-specific feeding environments by modifying the sensitivity of bitter taste receptors.

## Methods

### Construction of TAS2R expression vectors

Genomic DNA derived from blood samples of a captive common marmoset (*Callithrix jacchus*) and a captive Azara’s owl monkey (*Aotus azarae*) were provided by the Cooperative Research Program of the Primate Research Institute of Kyoto University under their Guidelines for the Care and Use of Laboratory Primates. Feces-derived genomic DNA of a wild white-faced capuchin (*Cebus capucinus*), a wild black-handed spider monkey (*Ateles geoffroyi*), and a wild mantled howler (*Alouatta palliata*) were obtained in previous studies by SK [11, 12]. *TAS2R1* and *TAS2R4* genes were cloned from these genome DNA samples using anthropoid genome-based sequences as PCR and sequencing primers [3].

TAS2R1 and TAS2R4 of marmosets, capuchin monkeys, owl monkeys, spider monkeys, and howler monkeys were tagged with the first 45 amino acids of rat somatostatin receptor 3 (sst tag) [13] and the last 8 amino acids of bovine rhodopsin (1D4 tag) [14] at the N- and C-terminal ends, respectively, and cloned into the mammalian expression vector pEAK10 (Edge BioSystems, Inc., Gaithersburg, MD, USA).

### Transfection of HEK293T cells

HEK293T cells were transfected with the TAS2R expression constructs together with the chimeric G protein subunit Gα16gust44 [15] in the mammalian expression vector pcDNA3.1 (Life Technologies, Inc., Carlsbad, CA, USA) using Lipofectamine 2000 (Life Technologies, Inc.). The empty pEAK10 vector was used as a negative control.

### Calcium assay

Calcium assays were performed as previously reported [4, 5], with some modifications. Briefly, one day after transfection, culture medium was exchanged with the assay buffer (10 mM HEPES, 130 mM NaCl, 10 mM glucose, 5 mM KCl, 2 mM CaCl_2_, 1.2 mM MgCl_2_, pH 7.4) and loaded with a calcium indicator, Calcium 4 (Molecular Devices, Inc., Eugene, OR, USA). Ligand solutions were prepared by dissolving bitter substances (camphor or colchicine) in the assay buffer and were added to cells. Fluorescence was measured at 525 nm following excitation at 485 nm with the FlexStation 3 Microplate Reader (Molecular Devices, Inc.). The calcium response amplitudes were expressed as ΔF/F, which is the ratio of the ligand-dependent increase in fluorescence to the fluorescence before the ligand addition. The response of cells that were transfected with the empty pEAK10 vector and Gα16gust44 was defined as the mock response (TAS2R-independent response) and subtracted from all responses.

### Ancestral sequence reconstruction

Ancestral amino acid sequences of NWM TAS2R1 and TAS2R4 were inferred using the maximum likelihood method implemented in MEGA5 [16, 17] with the Dayhoff and JTT [18, 19] amino acid substitution models [20]. The reconstructed ancestral TAS2Rs were tagged with sst and 1D4 tags at the N- and C-terminal regions, respectively, cloned into the pEAK10 vector, and expressed in HEK293T cells. Calcium assays were performed following the same protocol as that used for extant NWM TAS2Rs.

### Site-directed mutagenesis

Site-directed mutagenesis was performed using the QuikChange Multisite-Directed Mutagenesis Kit (Agilent Technologies, Inc., Santa Clara, CA, USA).

## Results

Single nucleotide polymorphisms were detected in *TAS2R1*of the capuchin monkey, and the spider monkey samples, and in *TAS2R4* of the mantled howler monkey sample (Additional file 1: Figure S1). Because there were no significant difference between haplotypes subjected to calcium imaging assays to explore the intraspecific variation in ligand sensitivity, we used representative haplotypes for each species to explore the interspecific variation.

**Fig. 1:**
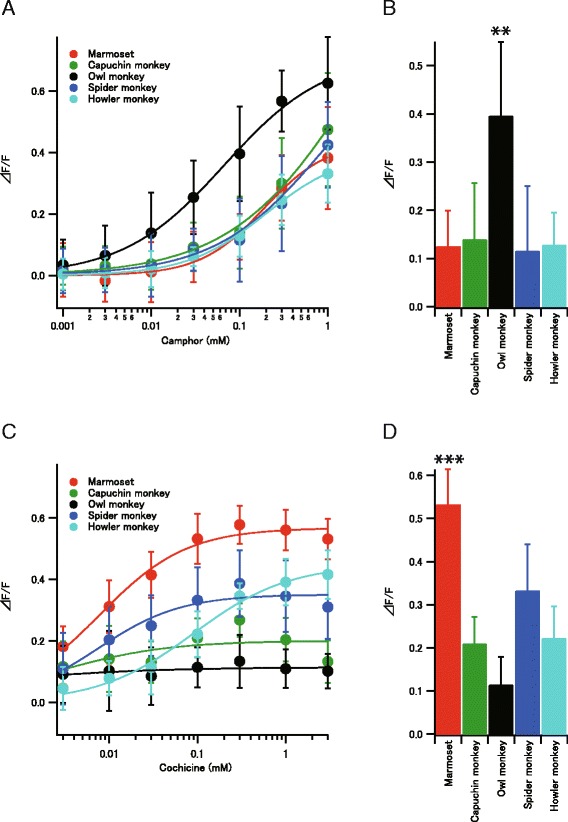
Responses of New World monkey (NWM) TAS1R and TAS2R4 to bitter substances. **a** Responses of TAS2R1s to camphor. TAS2R1s of the marmoset, capuchin monkey, owl monkey, spider monkey, and howler monkey were expressed in HEK293T cells and their responses to camphor were measured using calcium assays. Values are means with error bars representing standard deviations, which were calculated from the data obtained from at least eight experiments. **b** Response amplitudes of NWM TAS2R1s to 0.1 mM camphor. The owl monkey showed significant differences from any of the other species (pairwise comparisons using two-sided Welch's *t*-test with Benjamini-Hochberg correction, **p < 0.01). Any of the other pairs do not show significant differences (*p* > 0.05). **c** Responses of TAS2R4 to colchicine. TAS2R4s of the marmoset, capuchin monkey, owl monkey, spider monkey, and howler monkey were expressed in HEK293T cells and their responses to colchicine were measured using calcium assays. Values are means with error bars representing standard deviations, which were calculated from the data obtained from at least eight experiments. **d** Response amplitudes of NWM TAS2R4s to 0.1 mM colchicine. The marmoset showed significant differences from any of the other species (pairwise comparisons using two-sided Welch’s *t*-test with Benjamini-Hochberg correction, ***p < 0.001). Any of the other pairs do not show strong differences (*p >* 0.001)

We tested bitter substances that activate human TAS2R1 or TAS2R4 [4] for NWM TAS2R1 and TAS2R4. Among them, camphor and colchicine elicited detectable responses in NWM TAS2R1 and TAS2R4, respectively (Fig. 1). However, as the ligand concentration increased, the maximum response amplitude was not reached due to limitations in the solubility of ligands and cellular tolerance. Nevertheless, we detected clear variation in the amplitude of the response among species for both TAS2R1 and TAS2R4. Specifically, owl monkey TAS2R1 exhibited a significantly higher peak amplitude in response to camphor than the TAS2R1 orthologs of other NWMs (Fig. 1a), and marmoset TAS2R4 exhibited a significantly higher amplitude in response to colchicine than the TAS2R4 orthologs of other NWMs (Fig. 1c). Figure 1b and d show a comparison of the amplitude of the response using 0.1 mM of camphor and colchicine, respectively, among species. For TAS2R4, the EC50 value for the howler monkey (0.080 mM) was higher than those of other species (e.g., 0.067 mM for marmoset). We additionally tested other reported ligands for human TAS2R1 (~0.8 mM yohimbine, ~0.46 mM picrotoxinin, ~1 mM chloramphenicol, ~0.5 mM Thiamine) and TAS2R4 (~2 mM denatonium benzoate, ~2 mM diphenidol). However, these compounds generated too little response to analyze the differences among species.

**Fig. 2:**
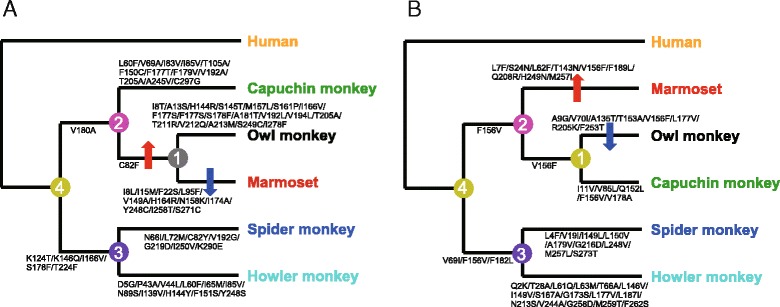
Phylogenetic relationships among extant and ancestral TAS2R1 and TAS2R4 receptors. **a** Phylogenetic relationships among extant and ancestral TAS2R1 receptors. The colors of the ancestral nodes 1–4 correspond to those in the graphs of Fig. 3. Amino acid substitutions are indicated along each branch. The arrows indicate increased/decreased camphor sensitivity. **b** Phylogenetic relationships among extant and ancestral TAS2R4 receptors. The colors of the ancestral nodes 1–4 correspond to those in the graphs of Fig. 3. Amino acid substitutions are indicated along each branch. The arrows indicate increased/decreased colchicine sensitivity

To estimate when the differences among species in ligand sensitivity for NWM TAS2R1 and TAS2R4 emerged, we inferred the amino acid sequences that are ancestral to extant NWM TAS2R1 and TAS2R4 (Fig. 2). Based on the phylogeny of the five sequences of NWM TAS2R1 or TAS2R4, ancestral nodes 1–4 were assigned to the tree topology and the ancestral amino acid sequences at these nodes were inferred. Amino acid substitutions were mapped on the branches of the tree (Fig. 2). The phylogenetic relationships among the marmoset, capuchin monkey, and owl monkey are still controversial [21], so three possible phylogenies (phylogenies 1, 2, and 3 in Additional file 2: Figure S2) were assumed and the sequences of ancestors 1–4 were inferred for each phylogeny. The phylogenies “2” and “3” were adopted for the phylogenetic relationship of NWM TAS2R1 and TAS2R4, respectively, based on the gene tree inferred using the neighbor-joining and maximum likelihood methods (Additional file 2: Figure S2).

**Fig. 3:**
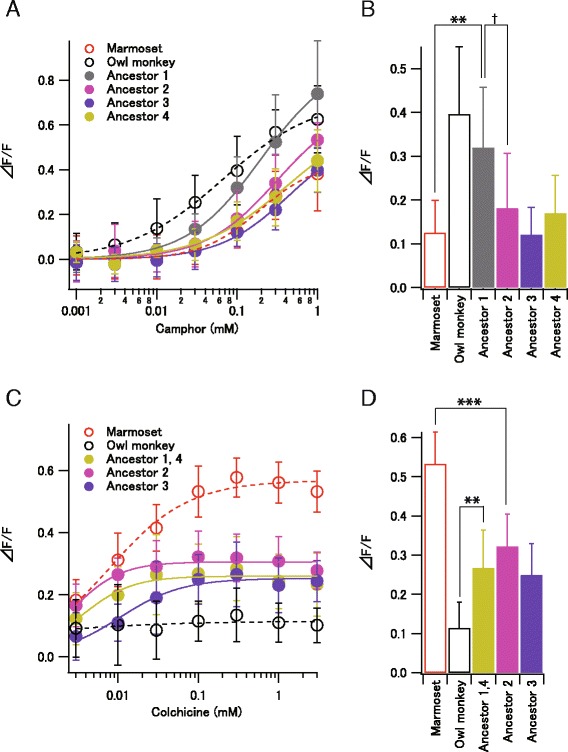
Responses of ancestral TAS2R1 and TAS2R4 receptors. **a** Responses of ancestral TAS2R1s to camphor. TAS2R1s of ancestors 1, 2, 3, and 4 (Fig. 2a) were expressed in HEK293T cells and their responses to camphor were measured using calcium assays. **b** Response amplitudes of ancestral TAS2R1s to 0.1 mM camphor. **p < 0.01,†*p* < 0.10, two-sided Welch’s *t*-test). Any of the node pairs in Fig. 2a do not show significant differences (*p* > 0.10). **c** Responses of ancestral TAS2R4 to colchicine. TAS2R4s of ancestors 1(4), 2, and 3 (Fig. 2b) were expressed in HEK293T cells and their responses to colchicine were measured using calcium assays. Because ancestors 1 and 4 have the same sequence, they were grouped together in Fig. 3c and d. **d** Response amplitudes of ancestral TAS2R4s to 0.1 mM colchicine. ***p < 0.001, **p < 0.01, two-sided Welch’s *t*-test). Any of blanches in Fig. 2b do not show significant differences between the nodes (*p* > 0.10)

We expressed the ancestral receptors with the inferred amino acid sequences in HEK293T cells and measured the responses to camphor or colchicine (Fig. 3). All of the ancestral TAS2R1 receptors, except “ancestor 1,” exhibited relatively low sensitivity to camphor, comparable to that of the TAS2R1 orthologs of the marmoset, capuchin monkey, spider monkey, and howler monkey. “Ancestor 1” had a marginally significantly higher sensitivity to camphor than “ancestor 2,” suggesting that the high sensitivity to camphor was acquired on the lineage from “ancestor 2” to “ancestor 1.” The sensitivity of “ancestor 1” to camphor was significantly higher than that of the marmoset, indicating that the sensitivity of TAS2R1 to camphor was reduced during evolution from “ancestor 1” to the marmoset (Fig. 2a).

All of the ancestral TAS2R4 receptors exhibited relatively low sensitivity to colchicine, comparable to that of TAS2R4 of the capuchin monkey, spider monkey, and howler monkey. However, they had significantly lower sensitivities than that of marmoset TAS2R4. Specifically, “ancestor 2” had a significantly lower sensitivity than that of marmoset TAS2R4, indicating that the high sensitivity of TAS2R4 to colchicine was acquired during evolution from “ancestor 2” to the marmoset. The sensitivity of “ancestor 1” was significantly higher than that of the owl monkey TAS2R4, indicating that the sensitivity was reduced during the evolution from “ancestor 1” to the owl monkey (Fig. 2b).

Candidate amino acid residues responsible for the evolutionary shifts in ligand sensitivity of NWM TAS2R1 and TAS2R4 can be detected. Only one amino acid residue differed between “ancestor 2” and “ancestor 1” of TAS2R4, position 82, which was occupied by a cysteine in “ancestor 2” and replaced with phenylalanine in “ancestor 1” (Fig. 2a). This indicates that the C82F replacement is responsible for the increase in ligand sensitivity from “ancestor 2” to “ancestor 1.” When we compared the amino acid sequence of the marmoset TAS2R1 with that of “ancestor 1,” we detected 11 substitutions (I8L, I15M, F22S, L95F, V149A, H164R, N158K, I174A, Y248C, I258T, S271C), one or some of which may be responsible for the reduction in the ligand sensitivity of TAS2R1 from “ancestor 1” to the marmoset. Similarly, the candidate amino acid residues responsible for the shifts in the ligand sensitivity of TAS2R4 can also be detected; L7F, S24N, L62F, T143N, V156F, F189L, Q208R, H249N, or M257L may explain the increase from “ancestor 2” to the marmoset and A9G, V70I, A135T, T153A, V156F, L177V, R205K, or F253T may explain the reduction from “ancestor 1” to the owl monkey.

**Fig. 4:**
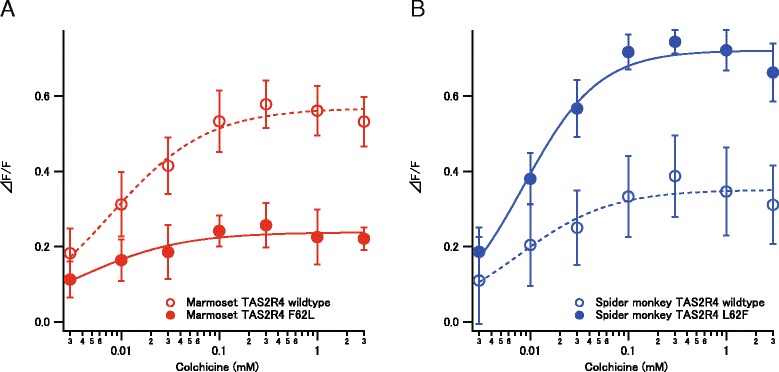
Site-directed mutagenesis experiments. **a** Response of the F62L mutant of marmoset TAS2R4 to colchicine. **b** Response of the L62F mutant of spider monkey TAS2R4 to colchicine

To identify the amino acid residue responsible for the differences in sensitivity among TAS2R4 orthologs of different species, we performed site-directed mutagenesis at a candidate site. When phenylalanine at position 62 in marmoset TAS2R4 was mutated to leucine, the response amplitude to colchicine was reduced (Fig. 4a). When leucine at position 62 in spider monkey TAS2R4 was replaced with phenylalanine, the amplitude of the response increased (Fig. 4b). These results show that the amino acid difference at position 62 is responsible for at least some of the differences in the response amplitude between TAS2R4 orthologs of marmosets and other species.

## Discussion

We measured the responses of NWM TAS2R1 and TAS2R4 receptors to bitter substances to explore variation in ligand sensitivity among extant species and reconstructed ancestors. We detected variation in the sensitivity of TAS2R1 and TAS2R4 to camphor or colchicine, respectively. TAS2R1 of the owl monkey and TAS2R4 of the marmoset exhibited significantly higher response amplitudes than the corresponding TAS2R1 and TAS2R4 receptors in other species (Fig. 1). Importantly, our results demonstrate how an ancestral receptor that is intermediately responsive to a variety of compounds can be shaped by lineage-specific evolution to respond more strongly to different compounds in different extant taxa.

The ecological implications of the variation in ligand sensitivity among species suggest that functional variation in compounds of dietary items or antifeedants are shaping the taste receptor genes of primates. Our findings emphasize the importance of investigating the role of bitter substances in the diets of NWMs. Recent studies have suggested the presence of TAS2Rs not only in the tongue, but also in other organs, like the nasal cavity and airway [2]. These receptors might therefore have a role in the detection of chemical compounds like camphor and colchicine based on their bitter taste, but also via other senses, such as olfaction.

Based on an analysis of ancestral receptors, we identified candidate amino acid substitutions responsible for the evolutionary shift in ligand sensitivity among species (Fig. 2). Specifically, we detected the following substitutions: C82F for the increase between “Ancestor 2” and “Ancestor 1” of TAS2R1; I8T, A13S, H144R, S145T, M157L, S161P, I166V, F177S, S178F, A181T, V192L, V194L, T205A, S249C, and I278F for the increase between “Ancestor 1” and owl monkey TAS2R1; I8L, I15M, F22S, L95F, V149A, H164R, N158K, I174A, Y248C, I258T, and S271C for the decrease between “Ancestor 1” and marmoset TAS2R1; L7F, S24N, L62F, T143N, V156F, F189L, Q208R, H249N, and M257L for the increase between “Ancestor 2” and marmoset TAS2R4; A9G, V70I, A135T, T153A, V156F, L177V, R205K, and F253T for the decrease between “Ancestor 1” and owl monkey TAS2R4. Homology modeling based on rhodopsin structures (or a recently resolved smoothened structure [22]) as a template would help reveal the mechanism underlying the interspecific variation. Additionally, site-directed mutagenesis experiments for these candidate residues are needed to identify the precise amino acid residues responsible for the observed interspecific variation in ligand sensitivity. Towards this goal, we conducted site-directed mutagenesis experiments and found that the amino acid difference at position 62 is one of the responsible sites for the difference in response amplitude between TAS2R4 of marmoset and other species. Because amino acid residue at position 62 would be situated in the extracellular side of second transmembrane domain [23], it might affect the ligand binding reaction of TAS2R4. Further investigations of additional candidate mutations may explain receptor function differences between NWMs.

## Conclusions

In summary, we compared the ligand sensitivity of the bitter taste receptors of NWMs. We found that sensitivity of TAS2Rs varies among species. These results raise the possibility that variation among TAS2Rs in ligand sensitivity is associated with ecological adaptations. Using an ancestral analysis, we detected evolutionary shifts in ligand sensitivity during the molecular evolution of TAS2R1 and TAS2R4, and we identified amino acid residues responsible for the shifts. Most of the ancestral receptors showed intermediate responsiveness between the most and least responsive existing receptors (i.e., TAS2R1: owl monkey and other species, respectively; TAS2R4: marmoset and owl monkey, respectively). Interestingly, these results suggest that ancestral receptors, characterized by intermediate responsiveness, evolved lower or higher responsiveness over time, as shown in the extant study species. Molecular and environmental studies will provide the basis for elucidating these phenomena and, more broadly, the diversification of NWMs, from molecular and ecological perspectives.

## Additional files

Additional file 1: Figure S1. Sequence alignment of extant and ancestral TAS2R1 and TAS2R4 of New World monkeys (NWMs). (A) Sequence alignment of extant and ancestral NWM TAS2R1 receptors. (B) Sequence alignment of extant and ancestral NWM TAS2R4 receptors. Abbreviations of the species names for sequences are as follows: Cj, common marmoset (Callithrix jacchus); Cc, white-faced capuchin (Cebus capucinus); Aa, Azara's owl monkey (Aotus azarae); Ag, black-handed spider monkey (Ateles geoffroyi); Ap, mantled howler (Alouatta palliata). (DOCX 742 kb)
Additional file 2: Figure S2. Reconstruction of ancestral TAS2R1s (A) and TAS2R4s (B). Ancestral amino acid sequences of New World Monkey TAS2R1 and TAS2R4 were inferred using the maximum likelihood method implemented in MEGA5 with the Dayhoff and JTT amino acid substitution models (Tamura K, Peterson D, Peterson N, Stecher G, Nei M, Kumar S. Mol Biol Evol 2011; 28(10):2731-2739). (DOCX 237 kb)
Additional file 3: Table S1. Original statistical data for calcium assays. (XLSX 243 kb)
