# Supplementary material for: Variation in ligand responses of the bitter taste receptors TAS2R1 and TAS2R4 among New World monkeys
Source: BMC Evol Biol. 2016 Oct 12;16:208. doi: 10.1186/s12862-016-0783-0 (PMC5062938; doi:10.1186/s12862-016-0783-0)
Supplement: Additional file 1: Figure S1. — Sequence alignment of extant and ancestral TAS2R1 and TAS2R4 of New World monkeys (NWMs). (A) Sequence alignment of extant and ancestral NWM TAS2R1 receptors. (B) Sequence alignment of extant and ancestral NWM TAS2R4 receptors. Abbreviations of the species names for sequences are as follows: Cj, common marmoset (Callithrix jacchus); Cc, white-faced capuchin (Cebus capucinus); Aa, Azara's owl monkey (Aotus azarae); Ag, black-handed spider monkey (Ateles geoffroyi); Ap, mantled howler (Alouatta palliata). (DOCX 742 kb) [file 12862_2016_783_MOESM1_ESM.docx]

(A)

(B)

**Figure S1.** Sequence alignment of extant and ancestral TAS2R1 and TAS2R4 of New World monkeys (NWMs).

(A) Sequence alignment of extant and ancestral NWM TAS2R1 receptors.

(B) Sequence alignment of extant and ancestral NWM TAS2R4 receptors.

Abbreviations of the species names for sequences are as follows: Cj, common marmoset (*Callithrix jacchus*); Cc, white-faced capuchin (*Cebus capucinus*); Aa, Azara's owl monkey (*Aotus azarae*); Ag, black-handed spider monkey (*Ateles geoffroyi*); Ap, mantled howler (*Alouatta palliata*).
