# Supplementary material for: Variation in ligand responses of the bitter taste receptors TAS2R1 and TAS2R4 among New World monkeys
Source: BMC Evol Biol. 2016 Oct 12;16:208. doi: 10.1186/s12862-016-0783-0 (PMC5062938; doi:10.1186/s12862-016-0783-0)
Supplement: Additional file 2: Figure S2. — Reconstruction of ancestral TAS2R1s (A) and TAS2R4s (B). Ancestral amino acid sequences of New World Monkey TAS2R1 and TAS2R4 were inferred using the maximum likelihood method implemented in MEGA5 with the Dayhoff and JTT amino acid substitution models (Tamura K, Peterson D, Peterson N, Stecher G, Nei M, Kumar S. Mol Biol Evol 2011; 28(10):2731-2739). (DOCX 237 kb) [file 12862_2016_783_MOESM2_ESM.docx]

(B)

**Figure S2.** Reconstruction of ancestral TAS2R1s (A) and TAS2R4s (B).

Ancestral amino acid sequences of New World Monkey TAS2R1 and TAS2R4 were inferred using the maximum likelihood method implemented in MEGA5 with the Dayhoff and JTT amino acid substitution models (Tamura K, Peterson D, Peterson N, Stecher G, Nei M, Kumar S. Mol Biol Evol 2011; 28(10):2731-2739).
